# Large-scale transcriptome analysis in chickpea (*Cicer arietinum* L.), an orphan legume crop of the semi-arid tropics of Asia and Africa

**DOI:** 10.1111/j.1467-7652.2011.00625.x

**Published:** 2011-10

**Authors:** Pavana J Hiremath, Andrew Farmer, Steven B Cannon, Jimmy Woodward, Himabindu Kudapa, Reetu Tuteja, Ashish Kumar, Amindala BhanuPrakash, Benjamin Mulaosmanovic, Neha Gujaria, Laxmanan Krishnamurthy, Pooran M Gaur, Polavarapu B KaviKishor, Trushar Shah, Ramamurthy Srinivasan, Marc Lohse, Yongli Xiao, Christopher D Town, Douglas R Cook, Gregory D May, Rajeev K Varshney

**Affiliations:** 1 International Crops Research Institute for the Semi-Arid Tropics (ICRISAT)Patancheru, India; 2 Osmania University (OU)Hyderabad, India; 3 National Centre for Genome Resources (NCGR)Santa Fe, NM, USA; 4 United States Department of Agriculture-Agricultural Research Service, Corn Insects and Crop Genetics Research Unit (USDA-ARS-CICGRU)Ames, IA, USA; 5 Department of Agronomy, Iowa State UniversityAmes, IA, USA; 6 National Research Centre on Plant Biotechnology (NRCPB), IARI CampusNew Delhi, India; 7 Max Planck Institute for Molecular Plant Physiology (MPIMPP)Am Muehlenberg, Potsdam-Golm, Germany; 8 J. Craig Venter Institute (JCVI)Rockville, MD, USA; 9 University of CaliforniaDavis (UC-Davis), CA, USA; 10 Generation Challenge Program (GCP)c/o CIMMYT, Mexico DF, Mexico

**Keywords:** chickpea, next generation sequencing, transcriptome, drought-responsive genes, markers

## Abstract

Chickpea (*Cicer arietinum* L.) is an important legume crop in the semi-arid regions of Asia and Africa. Gains in crop productivity have been low however, particularly because of biotic and abiotic stresses. To help enhance crop productivity using molecular breeding techniques, next generation sequencing technologies such as Roche/454 and Illumina/Solexa were used to determine the sequence of most gene transcripts and to identify drought-responsive genes and gene-based molecular markers. A total of 103 215 tentative unique sequences (TUSs) have been produced from 435 018 Roche/454 reads and 21 491 Sanger expressed sequence tags (ESTs). Putative functions were determined for 49 437 (47.8%) of the TUSs, and gene ontology assignments were determined for 20 634 (41.7%) of the TUSs. Comparison of the chickpea TUSs with the *Medicago truncatula* genome assembly (Mt 3.5.1 build) resulted in 42 141 aligned TUSs with putative gene structures (including 39 281 predicted intron/splice junctions). Alignment of ∼37 million Illumina/Solexa tags generated from drought-challenged root tissues of two chickpea genotypes against the TUSs identified 44 639 differentially expressed TUSs. The TUSs were also used to identify a diverse set of markers, including 728 simple sequence repeats (SSRs), 495 single nucleotide polymorphisms (SNPs), 387 conserved orthologous sequence (COS) markers, and 2088 intron-spanning region (ISR) markers. This resource will be useful for basic and applied research for genome analysis and crop improvement in chickpea.

## Introduction

Chickpea *(Cicer arietinum* L.) is of considerable agricultural importance. Grown on ∼11 million hectares, often as a dryland crop with few inputs, chickpea is particularly important for resource-poor farming communities of Asia and Africa (http://www.icrisat.org/crop-chickpea.htm). As a leguminous crop, chickpea provides a rich source of nitrogen, enhancing the soil fertility and is a valuable source of human dietary protein.

Genetic resource development for molecular breeding is important for energizing crop improvement programmes. The genomic resources currently available for chickpea, as compared to other legume crops, are very limited. Recently, about 20 162 Sanger ESTs (Varshney *et al.*, 2009a) and 48 796 BAC (bacterial artificial chromosome) - end sequences (BESs) have become available as collaborative efforts of ICRISAT and UC-Davis, USA. In terms of DNA-based molecular markers, about 2000 simple sequence repeat (SSR) markers are available (Hüttel *et al.*, 1999; Winter *et al.*, 1999; Lichtenzveig *et al.*, 2005; Sethy *et al.*, 2006; Nayak *et al.*, 2010). Additionally, 80 238 chickpea sequence tags have been generated using whole genome transcription profiling technology such as SuperSAGE (Molina *et al.*, 2008). However, because of unavailability of a reference genome sequence, analysis of smaller SuperSAGE tags is quite challenging.

The advent of high-throughput next generation sequencing (NGS) technologies such as Roche/454, Illumina/Solexa and ABI/SOLiD has made it possible to generate genome resources at large scale and relatively low cost (Mardis, 2008, Varshney *et al.*, 2009b). These technologies have been effectively used to generate large-scale transcriptome data in several plant species such as *Arabidopsis* (Weber *et al.*, 2007), *Medicago* (Cheung *et al.*, 2006), maize (Emrich *et al.*, 2007), barley (Steuernagel *et al.*, 2009) and soybean (Deschamps *et al.*, 2010). With an objective to develop transcriptomic and genomic resources in chickpea, this study employed two NGS technologies: Roche/454 and Illumina/Solexa. The Roche/454 sequencing technology was carried out on normalized cDNA pools comprised of cDNAs from 22 different developmental stage tissues of the reference genotype ICC 4958 to develop a transcriptome assembly, while Illumina/Solexa sequencing was undertaken on RNAs isolated from drought-challenged roots of parental genotypes (ICC 4958 and ICC 1882) of a mapping population. The short transcript reads (STRs) generated by Roche/454 were analysed together with the Sanger ESTs available at the time of analysis. The resulting contigs and singletons, that represent majority of the genes expressed in chickpea, were termed as ‘tentative unique sequences’ (TUSs). The TUS dataset was analysed with the following objectives: (i) development of a transcriptome assembly of chickpea, (ii) structural and functional characterization of the chickpea transcriptome, (iii) identification of differentially expressed drought-responsive genes, (iv) aid the understanding of global transcriptome changes because of drought responses, (v) development of genic markers. In summary, this study is the largest report to date of chickpea genomic and transcriptomic resources and of transcriptome responses to drought.

## Results

### Generation and assembly of transcript reads

**Figure 1:**
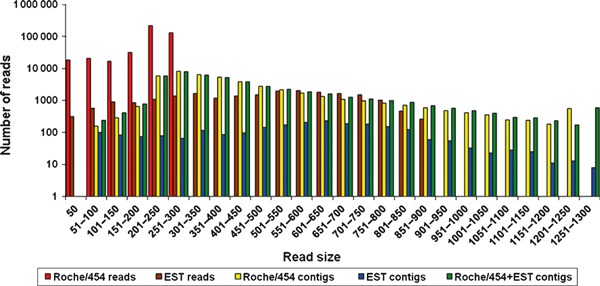
Read length distribution of Roche/454 reads and ESTs before and after assembly. Read size of Roche/454 sequences ranged from 50 bp to a maximum of 300 bp, with the highest number of reads having read size between 201 and 250 bp. Read size of high-quality ESTs varied from 50 to 900; maximum number of reads had 551–600 bp. A size comparison between raw Roche/454 reads and assembled Roche/454 reads (contigs) showed that majority of sequences in each case had size range between 201 and 300 bp, while similar comparison between raw ESTs and contigs showed a range of 551–600 bp. However, maximum of TUS contigs (12.67%) are ranged between 251 and 300 bp.

A normalized cDNA sample pool from chickpea cultivar ICC 4958, prepared from 22 tissues representing different developmental stages (embryo, shoots, roots, leaves, apical meristem, buds, flowers, young pods) of the plant, as well as challenged by abiotic stresses such as drought and salinity (details mentioned under ‘ Experimental procedure ’), was sequenced using the Roche/454 platform. A single sequencing run produced a total of 435 018 STRs with an average sequence length of 216 bp (Figure 1).

All 435 018 Roche/454 STRs were assembled using CAP3 (Huang and Madan, 1999). Post-assembly trimming of repetitive and poly-A sequence was performed using custom Perl scripts. Assembly of 435 018 Roche/454 reads produced 44 852 contigs and 87 806 singletons. Around 1704 contigs containing only two reads with zero read coverage variation were categorized as ‘high-confidence singletons’, because they might informatically represent unique genes expressed at low levels similar to singletons. The length of assembled contigs ranged from 159 to 650 bp with an average of 550 bp (Figure 1). A maximum number of contigs had a size range of 250–450 bp. Overall, the size of the contigs ranged from 100 to 1250 bp.

Assembly of Roche/454 STRs and Sanger ESTs together provided 44 845 contigs and 58 370 singletons, including 1679 ‘high-confidence singletons’. Thus, a total of 103 215 tentative unique sequences (TUSs) have been defined and will be referred to as the ‘chickpea transcriptome assembly’ (CaTA). The size of TUSs ranged from 54 to 3346 bp, with an average read length of 459 bp (Figure 1). The highest number of contigs (19 901) had a size range of 250–450 bp. Subsequently, this 103 215 TUS dataset was used for analysing the chickpea transcriptome for both gene structures and functions. Based on the CAP3 results it was observed that of the 21 491 Sanger ESTs that were used for analysis, 15 905 (74.1%) had similarity with 454 STRs while 5586 (25.9%) Sanger ESTs did not show any match and hence remained as singletons.

### Analysis with chickpea genomic survey sequences (GSSs)

The 103 215 TUSs were compared with 48 796 chickpea GSSs (NCBI, 20 October 2009). However, only 8218 (7.96%) TUSs showed significant matches to 4641 (9.51%) chickpea GSSs (≤1E-10, minimum query length of 70, and 80% identity). This indicates that the matched GSSs may be derived from coding regions (which may also include intronic or other noncoding regions).

### Comparison with the *Medicago* genome

As the closest legume model for chickpea is *Medicago truncatula*, we aligned the TUSs with the *Medicago* genome (Mt 3.5.1; http://www.medicagohapmap.org/?genome) to investigate gene coverage and gene structures. Taking into account the estimated time of divergence between chickpea and *Medicago*, as well as the error-prone nature of EST data, we used the HMM-based alignment program Exonerate (Slater and Birney, 2005), with thresholds requiring a minimum per cent identity of 75, a maximum intron length of 5000 bp, and retaining up to 10 alignments with Exonerate scores at least 50% as high as the top-scoring match. Out of 103 215 TUSs, 42 141 (40.8%) of the TUSs aligned with the *Medicago* genome, intersecting 14 580 predicted *Medicago* genes (Dataset S1). The alignments were also used to predict 39 281 splice sites in 20 137 of the TUS alignments and, furthermore, to predict intron-spanning primer sets. These alignments and primer sets are visible as a GBrowse track at the Legume Information System (LIS), at http://medtr.comparative-legumes.org/gb2/gbrowse/3.5.1/. The counts of best TUS alignments to *Medicago* chromosomes 1–8, respectively, were 4964, 4829, 5918, 6507, 6684, 1395, 5371 and 4201. The low count of alignments on chromosome 6 is noteworthy though not surprising, as this chromosome is known to be short and unusually repeat-dense (Cannon *et al.*, 2006).

### Functional annotation, categorization according to Gene Ontology (GO) descriptions

**Figure 2:**
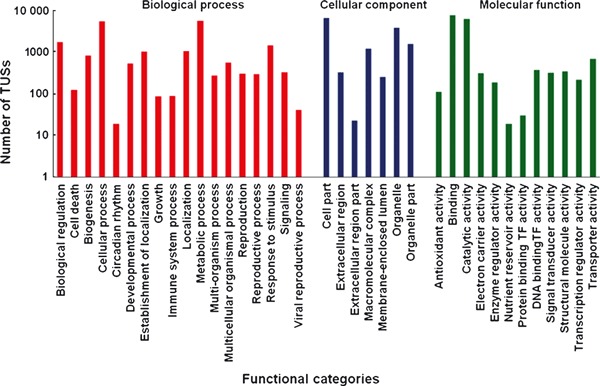
Functional categorization of chickpea TUSs. Chickpea TUSs (≤1E-10) were categorized hierarchically according to three principal gene ontologies, viz. biological processes, molecular functions and cellular components. Binding (46.35%) and catalytic activity (37.92%) subcategories of molecular function, organelle (28.17%) and cell part (47.12%) of cellular component, and metabolic process (28.19%) and cellular process (27.62%) of biological process categories were in higher proportion.

Comparison of the TUSs against the sequences of UniProt database (Uniref50) showed that 60 330 (58.45%) of TUSs had similarity (Dataset S2). At a threshold of ≤1E−10, functional annotations could be retrieved only for 49 437 TUSs (47.8%). These were functionally categorized based on GO descriptions. As a result, 20 634 (19.9%) TUSs were assigned to three principal categories: molecular function (10 963 TUSs), biological process (8099 TUSs) and cellular component (6662 TUSs). The highest number of TUSs fell into metabolic process (5631 TUSs, 28.19%), followed by cell part (6505 TUSs, 47.12%), binding (7714 TUSs, 46.35%), catalytic activity (6310 TUSs, 37.92%), cellular process (5517 TUSs, 27.62%) and organelle (3889 TUSs, 28.17%) subcategories (Figure 2).

Gene ontology classifications were also used to identify the genes related to stress responses. A large number of TUSs (1456; 7.29%) was found under the ‘response to stimulus’ subcategory. Additionally, Enzyme Commission (EC) IDs were retrieved for chickpea TUSs with a maximum number belonging to the ‘transferases’ (728) enzyme class, 671 to ‘hydrolases’ and 474 to ‘oxido-reductases’.

**Figure 3:**
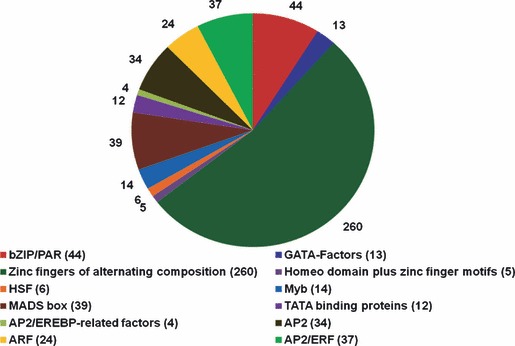
Transcription factors (TFs) identified by conserved domain annotation. Based on conserved domain characteristics, TUSs showing significant annotation to transcription factors were classified. Zinc fingers of alternating composition, MADS box and AP2/ERF were highly represented than other TFs.

Transcription factors (TFs) were identified from 49 437 TUSs based on conserved domains. TFs may be classified based on their (i) mechanistic, (ii) structural and (iii) functional properties. Within the mechanistic class, ubiquitous transcription factors such as TFIIB (six TUSs), TFIID (six TUSs), TFIIA (two TUSs) and TFIIE (one TUS) were identified. Structure-based classification is based on tertiary structures of DNA-binding domains, which are grouped under five super-classes which comprise various TF families (Figure 3). A total of 498 TFs were identified in the chickpea transcriptome: 44 of basic-helix-loop-helix class, 273 of zinc-coordinating DNA-binding class, 25 of helix-turn-helix class, 57 of β-scaffold factors with minor groove contacts, and 99 belonging to uncharacterized groups or from AP2 and ARF families.

### Gene expression analysis under drought conditions

Illumina/Solexa sequencing was performed on drought-challenged root tissues of two parental genotypes ICC 4958 and ICC 1882, to identify drought-responsive genes. As a result, 15.66 and 22.09 million tags (36 bp) were generated for ICC 4958 and ICC 1882, respectively. These reads were aligned against the TUSs using the ‘Alpheus’ pipeline of NCGR (Miller *et al.*, 2008) and used to identify differentially expressed genes. Expression data were available for 60 286 TUSs. Only 44 639 TUSs had a log difference value ranging between −4.5 and +4.3. The remaining 15 647 TUSs had expression values in only one of the libraries; subsequently, fold differences could not be calculated and hence were excluded from the analysis. Of the 44 639 TUSs, nine TUSs had more than a four-fold difference, 347 had three to four-fold difference, 2504 had two to threefold, 10 055 had one to two-fold and 31 724 had less than one-fold difference, while 9199 and 6448 were expressed exclusively in ICC 4958 and ICC 1882, respectively.

With an objective to display differentially expressed genes onto pathways and to obtain an overview of genes affected in response to drought in chickpea, the MapMan 3.0.0 tool was used on 44 639 genes for which differential expression values were available. The annotation tool ‘Mercator’ (http://mapman.gabipd.org/web/guest/app/mercator) allowed the assignment of 103 200 of 103 215 TUSs that were submitted, into a total of 36 functional classes, referred as BINs (Thimm *et al.*, 2004; Usadel *et al.*, 2009). Of these, 77 143 were classified as unknown or not assigned, while 26 057 were identified as belonging to known metabolic pathways or large enzyme families. The mapping file generated by the ‘Mercator’ pipeline was used for assigning differentially expressed chickpea TUSs obtained by comparing against five different databases (described in the Experimental procedure section).

The resulting mapping file was used to map the drought-responsive genes onto various pathways using the Image annotator module of the MapMan application. This allowed us to explore gene categories that are activated during drought response with more emphasis on those related to energy metabolism, secondary metabolism, transcription regulators and stress responses that are well documented to be responsive to wide-array of stresses. A total of 2974 TUSs [2860 TUSs which had greater than or equal to two-fold expression variation and also 116 TUSs with significant differential expression (*R* > 6) (Stekel *et al.*, 2000) excluding two common TUSs (Dataset S3)] were submitted to MapMan.

**Figure 4:**
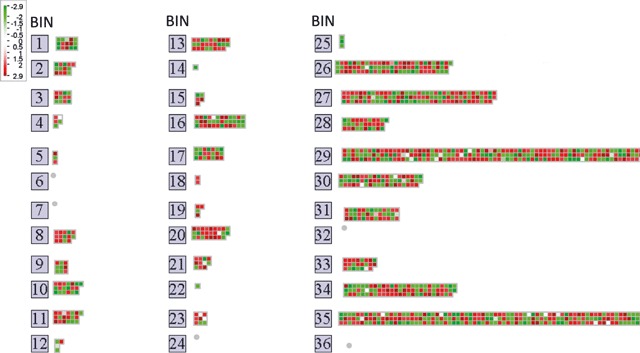
Overview of differentially regulated genes involved in different metabolic processes. Gene transcripts that are induced or repressed as a result of drought stress are shown in red and green colours respectively as shown in the colour bar ranging from −2.5 to +2.5. A total of 2823 TUSs out of 2974 genes related to various metabolic pathways were grouped under 31 BINs and were mapped using MapMan software to show the different functional categories involved. List of BINs are mentioned earlier by Thimm *et al.* (2004). List of the genes in each BIN is given in Dataset S4.

The overview map showed that a total of 2823 of 2974 differentially expressed TUSs/genes were mapped under 31 of 36 BINs (Figure 4, Dataset S4). While the majority of genes (1926 TUSs) were grouped in BIN 35 (‘not assigned’ category), the remaining 897 genes were assigned to 30 BINs. Of these, 583 (71.7%) genes belonged to six BINs and had higher proportion of genes comparatively, which include protein metabolism (BIN 29, 216 TUSs), RNA metabolism (BIN 27, 110 TUSs), miscellaneous enzyme families (BIN 26, 82 TUSs), transport (BIN 34, 80 TUSs), signalling (BIN 30, 59 TUSs) and cell (BIN 31, 39 TUSs).

Based on the assigned genes to different BINs, an attempt was made to understand differentially expressed genes of key metabolic reactions that often modulate normal cellular functioning during stress. As a result, energy metabolism [glycolysis, tri-carboxylic acid (TCA) cycle, electron transport chain (ETC) reactions], secondary metabolism, transcription factors and stress-related categories were analysed in detail, as follows:

#### Energy metabolism

Changes in the magnitudes of enzymes and metabolites of carbon and energy cycles have been identified to play crucial roles in cellular metabolism. Variations in glycolysis, TCA and ETC. cycles in response to stress are well documented for plants earlier (Apel and Hirt, 2004). The possible activation of respiratory activities in mitochondria and ATPs released during these reactions help to initiate tolerance events under hypoxic conditions (Kreuzwieser *et al.*, 2009). The genes (25 TUSs) related to energy metabolism belonging to BINs 4, 5, 6, 8 and 9 were identified in the chickpea transcriptome (Dataset S4). Genes coding for phospho fructokinase, pyruvate kinase, lactate dehydrogenase, pyruvate decarboxylase, etc. were induced (Figure S1a).

#### Secondary metabolism

Flavonoids and isoflavonoids are known to play a significant role in plant defence responses to pathogens (Dixon and Steele, 1999; Uppalapati *et al.*, 2009). Several genes (BIN 16) related to secondary metabolism such as phenylpropanoids (11 TUSs), terpenoids (three TUSs) and flavanoids (seven TUSs), which are expressed mainly in leguminous roots, were observed in response to stress, and hence, we expected their expression to be affected. Several genes involved in phenylpropanoid metabolism, such as phenyl ammonia lyase (*PAL*), coumarate:CoA ligase, cinnamoyl-CoA reductase family, putative/4-coumaroyl-CoA synthase and mannitol dehydrogenase, were observed in the study. Similarly, camelliol C synthase (*CAMS1*), terpene cyclase/mutase, beta-amyrin synthase of terpenoid and flavanoid pathways were also identified. All the aforementioned genes showed induction in sensitive ICC 1882 library as compared to the tolerant ICC 4958 library. Many other genes identified in this category are listed in Dataset S4 (Figure S1b).

#### Transcription factors

Expression of stress-responsive genes was shown to be regulated by two or more specific transcription factors present in the cell prior to stress (Srinivas and Swamynathan, 1996). Many genes (75 TUSs) assigned to transcription factors (BIN 27.3) (of several different TF classes) were identified and mapped. For instance, genes coding for Zinc finger family protein, MYB domain containing family, WRKY, auxin response factor, pentatricopeptide-repeat containing protein, bZIP were identified. A homologue to the early response to dehydration (ERD)-related protein of *Arabidopsis* was highly expressed in the drought susceptible library (562/million tags) as compared to the tolerant library (375/million tags). Genes belonging to this category showed highly individual responses in drought sensitive as well as tolerant libraries. However, overall, a clear trend in expression of all TFs together was not observed (Dataset S4 and Figure 4).

#### Stress genes

Molecular responses to stress factors such as heat shock, anaerobiosis, plant pathogens, oxygen free radicals, heavy metals, water stress and chilling in plants have been assessed in various plant species (Matters and Scandalios, 1986). In our study, 278 TUSs with stress-related annotations (either biotic or abiotic) were grouped (in BINs 10, 17, 20, 21, 26, 29 and 30). These included genes involved in red-ox reactions, cell wall breakdown, cell signalling and hormone signalling (Figure 4, Dataset S4 and Figure S1c). About 66% of TUSs (17/26) involved in abiotic stress (BIN 20.2) were found to be induced in tolerant ICC 4958 and repressed in sensitive ICC 1882, while the remaining 34% (9/26) TUSs showed the inverse pattern. The induced genes in ICC 4958 include abscisic acid-responsive protein (*ABR 17, ABR 18*), DNAJ heat shock protein, responsive to desiccation 22 (*RD22*), early ERD-related protein and various heat shock proteins (*HSP 70, HSP 91*). Interestingly, all genes encoding to ABRs were specifically induced in sensitive ICC 1882 library. These results are strongly conserved and are evidenced in earlier stress response studies (Swamy and Smith, 1999).

### Utilization of chickpea TUSs for development of molecular markers

With an objective to facilitate chickpea genetics and breeding, the TUSs were used for identification and development of several kinds of molecular markers, as described latter.

#### Identification and development of SSR markers

All TUSs (103 215) were mined for the presence of SSRs with the *MI*cro*SA*tellite (*MISA*) tool (Thiel *et al.*, 2003), giving 26 252 SSRs in 23 330 TUSs (Table 1). The most frequently occurring di-nucleotide motifs were AG followed by TC and CT, whereas among tri-nucleotides TTC is the highest.

**Table 1:** SSR identification using *MISA* search tool

Total number of TUSs examined	103 215
Total size of examined sequences (bp)	34 718 996
Total number of identified SSRs	26 252
Number of SSR containing sequences	23 330
Number of sequences containing >1 SSR	2480
Number of SSRs present in compound formation	2012
Mono-nucleotide repeats	24 428
Di-nucleotide repeats	743
Tri-nucleotide repeats	893
Tetra-nucleotide repeats	91
Penta-nucleotide repeats	51
Hexa-nucleotide repeats	46
Primer pair designed	3172
Class-I primer pairs selected for synthesis	728

*MISA*, *MI*cro*SA*tellite; SSR, simple sequence repeats, TUS, tentative unique sequence.

With an objective to convert the identified SSRs into potential genetic markers, an attempt was made to design the primer pairs for the TUSs containing SSR(s). Primer pairs could be designed for 3172 (12.08%) SSRs. Excluding the primer pairs for mono-nucleotide SSR motifs and for those yielding putative products of <100 bp, 807 primer pairs were considered suitable. All 807 TUSs were compared with the source sequences of SSR markers developed earlier (Hüttel *et al.*, 1999; Winter *et al.*, 1999; Lichtenzveig *et al.*, 2005; Sethy *et al.*, 2006; Nayak *et al.*, 2010) using BLASTN (Altschul *et al.*, 1990) at ≤1E-05, query coverage of ≥30 and per cent identity of >90, giving a set of 728 nonredundant primer pairs (Dataset S5).

To validate the newly designed EST-SSRs, a set of 80 primers (i.e. 16 from each informative SSR classes such as di-, tri-, tetra-, penta- and hexa-nucleotide) were randomly selected for synthesis and analysis. Of the 80 primer pairs that were screened, 71 showed amplification on five parental genotypes (ICC 4958, PI 489777, ICC 1882, ICC 283 and ICC 8261) of three chickpea mapping populations. While 42 SSR markers showed ≥2 alleles with a polymorphic information content (PIC) value ranging from 0.20 to 0.67 with a mean of 0.35, the remaining 29 markers amplified only one allele across five genotypes surveyed.

#### Conserved orthologous set (COS) markers

As mentioned earlier, 638 chickpea TUSs showed significant similarity with ESTs of all the six legume species (≤1E-30). Only 556 had an identical functional annotation, based on BLASTX (Altschul, 1993) (UniProt database, ≤1E-05) and across the legume species. Of the 556 TUSs, 90 TUSs were identified as potential paralogs and therefore a set of 466 TUSs were considered as putative orthologs. As another set of 1440 COS genes have already been developed at UC-Davis, USA (Douglas R. Cook, personal communication), the identified set of 466 TUSs in this study was analysed with 1440 COS genes. As a result, at ≤1E-05 and query coverage length of ≥25, 79 TUSs showed similarity with COS genes of UC-Davis and were subsequently excluded. Finally, the primer pairs were designed for a total of 387 nonredundant COS genes (Dataset S6).

#### Intron-spanning region (ISR) markers

Using the alignments of chickpea with the *Medicago* genome (Mt 3.5.1), ISR candidate markers were designed *in silico* for chickpea. These markers were designed from sequences having a single best match to the reference. A total of 2088 ISR primer pairs were designed across whole genome of chickpea (Dataset S7).

#### SNP identification based on Illumina/Solexa sequence reads

The utility of the TUSs was also demonstrated for SNP discovery. For this purpose, Illumina/Solexa sequences of ICC 4958 and ICC 1882 were aligned against TUSs using the ‘Alpheus’ program of NCGR (Miller *et al.*, 2008). A total of 26 082 potential nucleotide variants (transitions, transversions and indels) were identified between these two genotypes, using requirements of allele frequency (i.e. ratio of alleles at one locus observed among reads from another genotype) >0.1 and read depth ≥3 and <500 (Table 2). The number of likely, well-supported SNPs (e.g. 1503 SNPs with allele frequency ≥0.9 and coverage ≥3) was much smaller, consistent with generally low ranges of polymorphism in chickpea.

**Table 2:** Number of SNPs classified based on allele frequency and read depth

	Number of reads/tentative contigs			
	
Frequency difference range	>500	101–500	11–100	3–10
<0.1	389	751	2109	158
0.10–0.19	107	414	2431	500
0.20–0.29	17	123	3856	827
0.30–0.39	4	47	1478	992
0.40–0.49	1	13	746	828
0.50–0.59	8	18	502	1442
0.60–0.69	–	17	297	1361
0.70–0.79	–	1	85	374
0.80–0.89	–	–	55	166
0.90–1.0	–	–	40	1463

## Discussion

This study provides an extensive characterization of the chickpea transcriptome. For the first time, large-scale transcript sequence data were generated for identification of drought-responsive genes and development of gene-based markers to accelerate basic and applied genomics research in chickpea.

### Chickpea transcriptome characterization

The broad strategy of this project was to assemble a reference transcriptome assembly from a wide range of tissues, followed by genotype and stress-response comparisons using high-coverage short-read sequencing, and finally, development of several large marker resources. The gene diversity in the reference assembly benefitted from normalization of 22 pooled, diverse tissue libraries. Sanger reads (21 491) contributed to higher contig lengths, and Roche/454 reads (435 018) contributed coverage breadth and depth. This approach produced what we believe to be sequence coverage or sampling from the majority of chickpea genes, with 44 845 contigs and 58 370 singletons, and 459 bp average length for all TUSs. The assembly of Roche/454 reads produced relatively smaller size contigs (maximum size is 650 bp) as compared to contig assemblies (maximum size is 3346 bp) derived from clustering of Roche/454 and Sanger ESTs. This has been observed with CAP3 assembly data of Roche/454 data in other species also (Reinhardt *et al.*, 2009). The contigs size derived also depends on the assembler used (Kumar and Blaxter, 2010). Average per-base quality scores among different components of the Roche/454 reads is provided in Dataset S8. The Average read quality obtained for all the Roche/454 STRs generated in this study was ‘33′, which is considerably moderate. Also, the high probability of Roche/454 technology to miscall homopolymer lengths would be the main driver of CAP3 assembly problems and would be manifested as redundancy in the unigene set that was produced. In fact, a high level of redundancy was reflected in the alignments of the Solexa/Illumina reads to ‘CaTA’, with nearly half of all reads mapping equivalently to multiple regions on the reference. Without genomic assemblies, it is probably not feasible to rule out the possibility that this redundancy is biologically meaningful (e.g. because of recently duplicated gene families or to a large amount of splice isoforms), although it seems more likely that it represents an artefact induced by the assembler.

Sequence annotation of the chickpea TUSs based on BLASTX using nonredundant UniProt (Uniref 50) database showed significant functional annotation for approximately half (47.8%) of the TUSs. This less percentage of similarity observed may be partly because of the sequencing artefacts, lack of similarities available in UniProt databases and because of large number of unknown/hypothetical and uncharacterized sequence matches for newly identified genes in under-studied organisms like chickpea (Meyer *et al.*, 2009).

### Identification of drought-responsive genes

In this study, drought-responsive genes were identified using Illumina/Solexa 1G sequence data generated from drought-challenged root tissues of two parents (ICC 4958 and ICC 1882) that show distinct drought responses. The efficiency of Illumina/Solexa sequencing for the identification of differentially expressed genes has been well evidenced in a study by Hoen *et al.* (2008) in which the results obtained by Illumina/Solexa were compared with five different microarray platforms. As the sequence-based analysis does not require background correction as in microarray, cross-hybridization artefacts are avoided, low-abundant and rare genes may also be detected, and hence, the number of transcripts/genes analysed is comparatively greater than other technologies. Although sequence-based transcriptome expression analysis has great and broadly inclusive significance over other conventional techniques, it has a few challenges that include limitations of current sequencing costs, appropriate mapping of short reads on annotated regions and assignment of multi-mapping sequences, etc., but the improvements in massively parallel short-read sequencing chemistries and development of optimal algorithms for analysis will alleviate these challenges (Mortazavi *et al.*, 2008; Shendure, 2008).

We observed 2974 TUSs with significant expression changes, of which 2823 could be associated with gene ontology annotations. The chickpea transcriptome contained many genes encoding for aldehyde dehydrogenase, *O*-methyl transferases, naringenin-3-dioxygenase, oxido-reductases, farnesyl diphosphate synthetase, isopentenyl diphosphate isomerase, arogenate dehydrogenase, shikimate kinase related to secondary metabolism, energy metabolism and stress response. Their expression patterns do not suggest their co-regulation, but do point to activity in various secondary pathways (Dataset S4 and Figure S1a–c).

### Extending the repertoire of genic markers

Utilization of ESTs for large-scale gene discovery and marker development has been evidenced in many plants and crop species. This study resulted in several large new marker sets for chickpea, including SSRs, SNPs, COS and ISR primers. As these markers are derived directly from coding parts of the genome, they provide good opportunities to identify the ‘perfect marker’ for traits of interest.

EST/transcript-derived SSRs have been widely used in constructing high-density linkage maps, marker-trait association, diversity analysis, etc. in several crop species (Varshney *et al.*, 2002). As transcripts are more highly conserved than nongenic sequence, they are useful in detecting the signature of divergent selection (Li *et al.*, 2002). In this study, out of a total of 80 SSR markers that were validated, 71 (88.7%) of them showed scorable amplicons and nine markers (11.2%) did not yield any amplicons. Only 29 markers (36.2%) showed monomorphism and 41 markers (51.2%) showed occurrence of ≥2 alleles. This can be attributed to their high level of conservation (Varshney *et al.*, 2005).

COS markers have found wide application in cross-genome comparative studies in legume species. In a separate study, 1440 tentative orthologs genes (TOGs) have been identified for six legume species namely chickpea, pigeonpea, common bean, cowpea, groundnut and lentil (Douglas R. Cook, personal communication). This study produced a new set of 329 nonredundant COS markers chickpea that can be used for cross-legume species comparisons (Dataset S6).

Because of the use of SNPs in high-throughput genotyping, the SNP marker system is becoming very popular in plant genetics and breeding applications. This study provides the first large (26 082) set of potential SNPs in chickpea. However, using stringent criteria (>0.60 frequency range and >10 read depth), 495 high-confidence SNPs were identified. Conversion of these SNPs into assays such as GoldenGate (Rostoks *et al.*, 2006) or KASPar (http://www.kbioscience.co.uk) will provide a low-cost and high-throughput marker genotyping system for accelerating their use in genetics and breeding programmes.

## Conclusion

In summary, this study provides a large transcript dataset for chickpea and describes insights into the chickpea transcriptome and differential responses to drought. Development of about 3000 gene-based markers is another important output of this study that can readily be used to accelerate chickpea genetics and breeding applications.

## Experimental procedures

### Chickpea transcriptome assembly and RNA extraction

About 22 different tissues of the ICC 4958 chickpea genotype representing different developmental stages such as embryo, leaves, apical meristem, shoots, roots, buds, flowers, pods of the plants as well as drought and salinity stressed roots were harvested at different time points to maximize the diversity of expressed genes in our experimental material. List of different developmental stage tissues harvested is provided in Dataset S9. Total RNA was extracted from all the tissues using protocol of Schmitt *et al.* (1990).

### cDNA library construction and normalization

Total RNA samples were reverse transcribed to full-length enriched cDNA using the SMART approach (Zhu *et al.*, 2001). cDNAs were directionally cloned in a two-step reaction and normalized using outsourcing services of Evrogen (Moscow, Russia) (http://www.evrogen.com).

### Sequence screening and assembly

All the Roche/454 generated and Sanger ESTs were prescreened to remove adaptor-ligated regions and low-complexity homopolymer regions. Clustering and assembly of qualified Roche/454 reads and Sanger reads were performed using CAP3 (Huang and Madan, 1999). The following parameters were used for all CAP3 assemblies: -p 95 -o 50 -g 3 -y 50 -t 1000. These parameters were chosen to satisfy three primary goals: (i) to maximize contig length, (ii) to minimize production of contigs with highly variable read coverage, as these tend to be spurious assemblies, (iii) increasing the value of the ‘-t’ parameter improves the quality of the assembly at the cost of using additional memory on the assembly server; the value of ‘1000′ was chosen as it was higher than the default but remained within the memory constraints of the assembly server. TUSs thus derived were further used for downstream analyses. All those assemblies with only two reads, which are considered as contigs by CAP3, were categorized as high-confidence singleton reads.

### Sequence annotation

Similarity search for TUSs against the UniProt databases was performed to retrieve sequence annotations using standalone BLASTX algorithms considering an *E*-value cut-off of ≤1E−10.

### Mapping of sequences using Exonerate

Alignment of chickpea TUSs was performed against the Mt 3.5.1 genome build using Exonerate (Slater and Birney, 2005) with a maximum allowed intron size of 5000 bp, requiring ≥50% identity and retaining alignments with scores within 50% of the best-scoring alignment. For development of ISR markers, primer3 (Rosen and Skaletsky, 2000) and custom perl scripts were used to identify primers that flanked intron junctions. Two TUSs with ≤1000 intervening bp without transcript alignment coverage were considered likely to be part of the same transcribed unit or the gene.

### Illumina/Solexa sequencing of ICC 4958 and ICC 1882

Drought stressed (Polyethylene glycol induction, sudden dehydration, slow drought stress in green house and Slow drought stress in field) root samples of both ICC 4958 and ICC 1882 were harvested when the end point of transpiration ratio reached 0.1 (Varshney *et al.*, 2009a). Total RNA was extracted from all the stressed root samples of both the genotypes as mentioned earlier. Subsequently, RNAs collected from different samples of one genotype were pooled, and pooled RNA for each genotype was used for Solexa sequencing on Illumina’s Genome Analyzer I at NCGR, Santa Fe, NM, USA.

### Expression profiling and MapMan analyses

For identification of differentially expressed genes between drought stressed libraries of ICC 4958 and ICC 1882 genotypes, expression values were derived by counting the number of sequence tags that mapped to transcript assembly developed in this study. Data normalization for more precise quantification was performed by considering per million reads for calculating the expression values, because the number of tags mapped was slightly higher. The expressed values of differentially expressed genes/TUSs mentioned throughout the text are ≥log2 scale values, because a minimum of two-fold change value is required for a visible coloration on map. The data are exported to MapMan 3.0.0 tool (Thimm *et al.*, 2004; Usadel *et al.*, 2005) which converts the data values to colour scale. The transcripts not called are represented as grey, transcripts that change by less than threshold value of 0.5 are white, transcripts increased are red and transcripts decreased are in green.

Differentially expressed genes/TUSs between drought-responsive genotypes were identified based on R-Statistics (*R* > 6) and those with greater than two-fold expression values were considered. The logarithmic-based expression values of each significant gene were subtracted between the libraries of genotype pairs of both studies, thus leading to a ‘+’ value in case of above-average expression levels and a ‘−’ value in case of below-average expression levels. A MapMan BIN file with hierarchical ontology system for chickpea genes was prepared using Mercator (http://mapman.gabipd.org/web/guest/app/mercator) by comparing against already classified proteins. All TUSs were used for searches against five different databases: The Arabidopsis Information Resource (TAIR8) proteins (Swarbreck *et al.*, 2008), SwissProt/Uniprot plant proteins (Schneider *et al.*, 2005), Conserved Domain Database (CDD; Marchler-Bauer *et al.*, 2007), Clusters of Orthologous Groups (COG; Tatusov *et al.*, 2003) and InterProScan (Zdobnov and Apweiler, 2001). The programs used to perform the searches were BLASTP (Altschul *et al.,* 1990) for TAIR8 and PPAP and RPSBLAST (Schaffer *et al.*, 2001) for CDD and COG. Database hits with bit scores <80 were ignored as not significantly similar. The results of all searches were compiled into one table, and reference mappings of the above-listed databases were then used to assign preliminary MapMan BIN codes to each of the TUSs. In the next step, the bit scores (in the case of TAIR8, PPAP, CDD and COG) for each database hit were recorded and evaluated for sequence as a measure of reliability for the assignment of proteins into certain BINs. To finally assign the protein to BINS, the bit scores of all database hits belonging to the same BIN were combined, allowing for multiple assigned BIN codes. An experimental data file containing expression values for each gene are represented in rows and libraries as columns.

### EST-SSR identification, screening and data analysis

Identification of SSRs in TUSs was performed using *MISA* search tool (Thiel *et al.*, 2003). *MISA* search provides information about the type and localization of each individual microsatellite and parses the calculated primer sequences, their sequence and melting point, melting temperature and expected PCR product size. For assessing the potential of the newly developed EST-SSRs, the markers were amplified on five different chickpea genotypes (ICC 4958, ICC 1882, ICC 283, ICC 8261, PI 489777). Polymerase chain reaction (PCR) was performed as described earlier (Varshney *et al.*, 2009a). Data were analysed using GeneMapper® Software v4.0 (Life Technologies Corporation, Carlsbad, CA, USA). PIC value, and other marker informations were obtained using PowerMarker v3.25 (Liu and Muse, 2005).

## Supporting information

**Figure S1** (a) Mapping of differentially expressed chickpea TUSs of energy metabolism. Genes related to energy metabolism (glycolysis, TCA and ETC) were mapped. Up-regulated genes (greater than or equal to two-fold) in ICC 4958 as compared to ICC 1882 are represented in red. Detailed list of genes related to these pathways are provided in Dataset S4. (b) Mapping of differentially expressed genes onto secondary metabolism pathway related categories using MapMan. Around 48 genes related to secondary pathways such as shikimate pathway, MVA pathway, phenylpropanoid pathway, Flavanoid metabolism, etc. Detailed gene list given in SI Dataset S4. (c) Mapping of differentially genes related to stress response mapped. Overall 278 TUSs annotated to stress related reactions are shown. The map shows the genes which are elicited in response to both biotic and abiotic stress. The list of genes categorised under different BINs are given in Dataset S4.

**Dataset S1** Identification of splice-sites in chickpea.

**Dataset S2** BLASTX analysis results of chickpea TUSs against the UniProt database.

**Dataset S3** Significant and differentially expressed drought-responsive chickpea TUSs.

**Dataset S4** Mapping results of significantly differentially expressed chickpea TUSs using MapMan 3.0.0 tool.

**Dataset S5** Novel set of chickpea SSR primer pairs.

**Dataset S6** Novel set of conserved orthologous set (COS) primer pairs.

**Dataset S7** Intron spanning region (ISR) markers in chickpea.

**Dataset S8** Average per-base quality scores among different components of the Roche/454 dataset.

**Dataset S9** List of developmental stage tissue samples used for Roche/454 sequencing.
