# Supplementary figures and images for: Large-scale transcriptome analysis in chickpea (Cicer arietinum L.), an orphan legume crop of the semi-arid tropics of Asia and Africa
Source: Plant Biotechnol J. 2011 Oct;9(8):922–31. doi: 10.1111/j.1467-7652.2011.00625.x (PMC3437486; doi:10.1111/j.1467-7652.2011.00625.x)

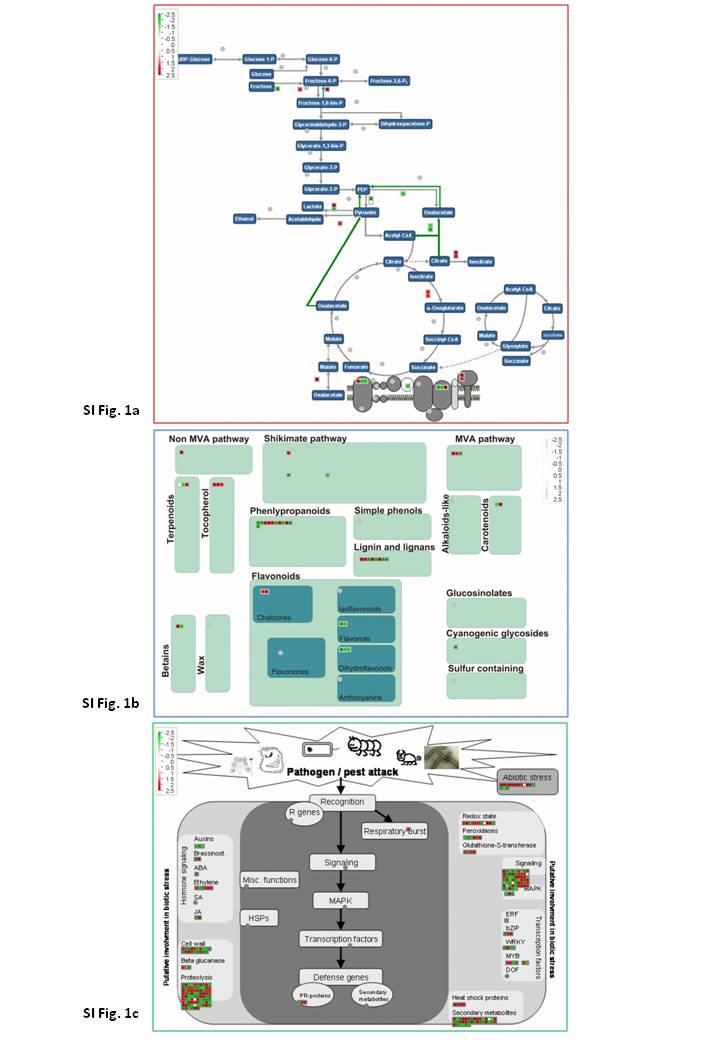

Supplement: Supplementary file 1 [file pbi0009-0922-SD1.jpg]
